# Housing materials as predictors of under-five mortality in Nigeria: evidence from 2013 demographic and health survey

**DOI:** 10.1186/s12887-016-0742-3

**Published:** 2017-01-19

**Authors:** Stephen Ayo Adebowale, Oyewale Mayowa Morakinyo, Godson Rowland Ana

**Affiliations:** 1 Department of Epidemiology and Medical Statistics, Faculty of Public Health, College of Medicine, University of Ibadan, Ibadan, Nigeria; 2 Department of Environmental Health Sciences, Faculty of Public Health, College of Medicine, University of Ibadan, Ibadan, Nigeria

**Keywords:** Housing materials, Under-five mortality, Nigeria

## Abstract

**Background:**

Nigeria is among countries with high Under-Five Mortality (U5M) rates worldwide. Both maternal and childhood factors have been linked to U5M in the country. However, despite the growing global recognition of the association between housing and quality of life, the role of housing materials as predictors of U5M remain largely unexplored in Nigeria. This study, therefore, investigated the relationship between housing materials and U5M in Nigeria.

**Methods:**

The study utilised the 2013 Nigeria Demographic and Health Survey data. A representative sample of 40,680 households was selected for the survey. The sample included 18,516 women of reproductive age who had given birth in the past 5 years prior the survey; with attention on the survival status of the index child (the most recent delivery). Data were analysed using descriptive statistics, Chi-square, Cox-proportional hazard and Brass 2-parameter models (α = 0.05).

**Results:**

The hazard ratio of U5M was 1.46 (C.I = 1.02–1.47, *p* < 0.001) and 1.23 (C.I = 1.24–1.71, *p* < 0.001) higher among children who lived in houses built with inadequate and moderate housing materials respectively than those in good housing materials. Under-five deaths show a downward trend (slope = −0.4871) relative to the housing materials assessment score. The refined U5M rate was 143.5, 127.0 and 90.8 per 1000 live birth among women who live in houses built with inadequate, moderate and adequate housing materials respectively. Other predictors of U5M were; the size of the child at birth, preceding birth interval, prenatal care provider, residence and education. Under-five death reduces with increasing maternal level of; education, wealth quintile, media exposure and housing material type and mostly experienced by Muslim women (6.0%), rural women (6.5%) and women residence in the North-West geopolitical zones (6.9%).

**Conclusions:**

Living in houses built with poor housing materials promoted U5M in Nigeria. Provision of sustainable housing by the government and the maintenance of existing housing stock to healthful conditions will play a significant role in reducing the burden of U5M in Nigeria.

## Background

Housing is a key determinant of health and quality of life [1]. Housing is designed to provide shelter and protection from hazards resulting from the physical and social environments. It promotes physical and mental health and ensures the social and economic well-being and quality of life of individuals and households [2]. A person’s home is an essential locus for everyday life [3]. Environmental living conditions, including housing conditions and their association with health have drawn the attention of public health scientists since ancient times [4].

Healthy housing has been defined as dwellings and premises that are built and maintained in ways that support the health of its occupants [5, 6]. In the year 1996, the United Nations Habitat conference that held in Istanbul defined healthy housing to include the provision of adequate physical, chemical, biological and mental conditions that supports health, comfort and privacy [7].

Researchers have established that good housing conditions are indispensable in improving household health. Poor housing and environmental conditions can predispose to adverse health problems, including infectious diseases (respiratory disorders), stress and depression [8]. The wellbeing of household occupants is a reflection of the building materials from which the house is built [9, 10]. One of the established ways through which housing influences health is by human exposure to poor housing conditions, lack of improved water sources, ineffectual waste disposal methods, invasion by disease vectors and inappropriate food storage techniques [11].

Deficient and worsening housing conditions can trigger a range of diseases, including lung diseases, neurological disorders, mental and behavioural dysfunction, which mostly unduly affect children [12]. Children are predominantly susceptible to housing-related hazards than adults since they spend comparably more time indoors. They are seen as a risk group because they require a higher amount of air inhalation than adults, and their organs are not fully developed [13, 14]. Children also have larger surface area to total body mass, thus causing increased exposure to pollutants [15].

Child mortality is a core indicator for measuring the level of child health and well-being in all nations [16]. It is an important indicator of the performance of the health system of a country [17]. Since the beginning of the twentieth century, childhood mortality has been at the centre of health discourse. Health professionals and policy makers have reserved a unique interest in combating the increasing childhood mortality rates [18]. This interest has not only extended globally, it has led to the development of logical approaches to reducing child mortality by two-thirds among children under the age of 5 years between 1990 and 2015, as tagged in the United Nation’s Millennium Development Goals (MDGs) [18]. With 133 of the 195 countries that adopted the MDGs failing in meeting the target of a two-thirds reduction in U5M rate, the United Nations in 2015 adopted the Sustainable Development Goals (SDGs) with the intention of safeguarding healthy lives and ensuring the well-being of all children. The goal 3 target 3.2 of the SDGs is to halt preventable deaths of newborns and under-fives by the year 2030 [19].

Under-five mortality rate is highest in sub-Sharan Africa with 1 child in 12 dying before 6 months, which translates to more than 12 times higher than the 1 in 147 average in developed countries [16]. India and Nigeria account for more than one-third of all under-five deaths globally [20]. Currently, Nigeria has the highest reported number of under-5 deaths in Africa [21] with about one million of them dying annually [22]. A number of factors are contributory to this high prevalence of under-five mortality in Nigeria [20, 23–32].

Provision of healthy housing and the continuous maintenance of existing housing stock in such a way as to support and promote human health remain a huge challenge in many countries [9, 33]. In sub-Saharan Africa more than 60% of urban dwellers live in slums [34], characterised with poor housing, insufficient space, unimproved water and sanitation. Though the association between living in healthy housing and good quality of life have been described in many developed nations, the links between housing and under-five mortality remain largely unexplored in developing nations [35].

While many studies and reports (including the 2013 report of the Nigeria Demographic and Health Survey) have indicated that the demographic characteristics of both mothers and children play an important role in a child survival [36], there is dearth of information on the role of housing materials in the causation of under-five child mortality while controlling for mother and child individual level factors. To this end, the present study seeks to advance the existing knowledge beyond the understanding of medical-related or personal characteristics by examining the influence of housing conditions and other environmental factors on under-five mortality in Nigeria.

## Methods

### Study area

The study was carried out in Nigeria, with a human population figure of above 180 million. Nigeria is characterised with high infant and childhood mortality. The maternal mortality ratio is also one of the highest among developing nations worldwide [36]. High fertility is a key factor for high under-five mortality across countries [37]. In Nigeria, the total fertility rate remains high (TFR = 5.5) [36]. Unfortunately, the contraceptive prevalence rate of 10% is still considered as low [36]. Nigeria is seen on the global page as poverty stricken country with a striking gap between the rich and the poor. Administratively, Nigeria is made up of 36 states and the Federal Capital territory. Each state has local government areas (third level of government) which are further divided into localities. The country is also stratified into 6 geopolitical zones; North-Central, North-East, North-West, South-East, South-South and South-West. The reason being that Nigeria comprised approximately 400 ethnic groups and 450 dialects. There was the need for the government to unify similar groups into zones for effective allocation of resources. The inhabitants in each of the geopolitical zones are homogeneous and share similar socio-cultural characteristics and unique in other health-related characteristics like access to health care, environment, housing system etc.

### Data collection

The Demographic and Health Surveys (DHS) and alike surveys that collect information on birth outcomes from women are one of the main bases for data collection on under-five mortality estimation in developing countries like Nigeria, where reliable and adequate vital registration system is lacking [17]. The 2013 Nigeria Demographic and Health Survey data were used for this study. The original data was collected to provide information on the population, health and fertility levels in Nigeria. A 3-stage stratified cluster design consisting of 904 clusters (372 in urban areas and 532 in rural areas) was used for sample selection. A representative sample of 40,680 households was selected for the survey and a fixed sample of 45 households was selected in each cluster. In this study, the sample used was 18,516 women of reproductive age who had given birth in the past 5 years prior the survey. However, the attention was focused on the survival status of the index child, the most recent delivery the women had in the past 5 years prior the study.

### Variable description

The dependent variable was childhood mortality and this was captured with the question on the survival status of the most recent birth (dead = 1 or alive = 0) in the last 5 years preceding the survey. Under-five mortality was defined as the death of a live-born child before its fifth birthday [27]. The main predictor was the type of housing materials. This was obtained as aggregate score based on information from roof materials (Improved – cement, roofing sheets, ceramic tiles; Unimproved – natural, no roof, palm leaf, sod, rudimentary, rustic mat, bamboo, cardboard), wall materials (Improved – cement, stone with cement, cement blocks, bricks; Unimproved – natural, no wall, palm/trunks, dirt, rudimentary, bamboo with mud) and floor materials (Improved – cement, ceramic tiles, vinyl asphalt strips, parquet, polished wood, finished; Unimproved – natural, earth, sand, dung, rudimentary, wood planks, palm, bamboo, others). The improved categories assumed a score of 1 while unimproved scored [36, 38]. The overall score (13-point maximum and 0-point minimum) for a woman was disaggregated into three categories: inadequate (<50% of the overall score), moderate (50% ≤ x < 75% of the overall score) and adequate (75% ≤ x ≤ 100%) of the overall score). They are based on quartile classification: 3rd quartile is 75% and 2nd quartile is 50%.

Other independent variables include mother’s age, highest educational level, religion, ethnicity, marital status, place of residence, region, wealth index and media exposure. others are; number of antenatal visits, tetanus injection, gender of a child, size at birth, birth order, preceding birth interval, prenatal care provider, delivery assistance, place of delivery, cooking fuel (Clean – electricity, liquefied petroleum gas, natural gas, biofuel; Unclean/biomass – coal, charcoal, wood, straw/shrubs/grass, agricultural crop, animal dung, kerosene), source of water (Improved - piped into dwelling, public tap, borehole, protected well and spring, rain water and bottle water; Unimproved – other sources not listed as improved sources) and toilet facility (Improved – flush/pour flush to piped sewer system, septic tank or pit latrine, ventilated improved pit latrine, pit latrine with slab, composting toilet; Unimproved – other toilet types not listed as improved). We adapted the groupings of environmental factors documented in the 2013 Nigeria National Demographic Health Survey (NDHS) and the 2010 WHO and UNICEF document on progress on sanitation and drinking water [36, 39]. These variables were used as covariates during multivariate analysis to determine the association between housing materials and childhood mortality.

### Data analyses

The dataset was weighted before data analyses. The weight is an inflation factor which extrapolates the sample to the target population [40]. Analysis of data was done at bivariate and multivariate levels using Chi-square, Cox proportional hazard model and Brass 2-parameter model. The Chi-square test was used to examine the relationship between child’s survival status and the independent variables at 5.0% level of significance. At the multivariate level of analysis, Cox proportional hazard model was used to detect the predictors of childhood mortality. The proportional hazards model assumes that the time to event and the covariates are related as; ${ \log}_{\mathrm{e}}\left\{\frac{\upgamma_{\mathrm{i}}\left(\mathrm{t}\right)}{\upgamma_0\left(\mathrm{t}\right)}\right\}={\upbeta}_0+{\upbeta}_1{\mathrm{x}}_{\mathrm{i}1}+{\upbeta}_2{\mathrm{x}}_{\mathrm{i}2}+\dots +{\upbeta}_{\mathrm{p}}{\mathrm{x}}_{\mathrm{i}\mathrm{p}}$. Where; γ_i_(t) is the hazard rate for the i^th^ case of a woman having lost her child before age five; γ_0_(t) is the baseline hazard at time t when the death of the child occurs; β_j_ is the value of the j^th^ regression coefficient; x_ij_ is the value of the i^th^ case of the j^th^ covariate.

Further analysis using an indirect method to ascertain the influence of housing materials on childhood mortality was done. Brass [41] reported that the probability of dying between birth and exact age (a) can be estimated as q(x) = k(x) × D(x) where D(x) is the number of dead children in each age group and k(x) is a multiplier and is estimated as; k(x) = a(i) + b(i)(P_1_/P_2_) + C(i)(P_2_/P_3_). The number of non-surviving children for women in age groups 15–20, 20–25, 25–30,…, 45–50 were used to calculate childhood mortality at exact ages (q(x)); 1, 2, 3, 5, 10, 15 and 20. Regression equations which relate the multipliers k(x) to indices of fertility schedule were formulated from mathematical simulations [42]. The time reference to which the q(x) values refer were also formulated. Due to limitations in the estimates produced by this method, we further adjusted the childhood mortality using Brass 1-parameter model (Y = ∝ + βY_s_) where β = 1. This procedure made use of logit equation: $\mathrm{logit}\left\{\mathrm{q}\left(\mathrm{x}\right)\right\}=\frac{1}{2} \log \left\{\frac{1-{\mathrm{l}}_{\mathrm{x}}}{{\mathrm{l}}_{\mathrm{x}}}\right\}$. This was transformed to Brass relational system of life tables; logit{l(x)} = ∝ + βlogit{l_s_(x)} usually transcribed as Y = ∝ + βY_s_. The logit relational system smoothens the estimated values of l(x) (survival probability) in contrast to the values from the model life-table. Therefore, if β = 1, $\widehat{\propto}=\mathrm{Y}\left(\mathrm{x}\right)-{\mathrm{Y}}_{\mathrm{s}}\left(\mathrm{x}\right)$, thus generating; $\mathrm{Y}(1)={\widehat{\propto}}_1+{\mathrm{Y}}_{\mathrm{s}}(1);\mathrm{Y}(2)={\widehat{\propto}}_2+{\mathrm{Y}}_{\mathrm{s}}(2);\dots; \mathrm{Y}(20)={\widehat{\propto}}_{20}+{\mathrm{Y}}_{\mathrm{s}}(20)$. Estimate of $\widehat{\propto}$ was obtained from the average values of x = 2, x = 3 and x = 5 which produce reliable values of l(x). Therefore, if $\overline{\mathrm{Y}}$ is the average of Y(2), Y(3) and Y(5) and ${\overline{\mathrm{Y}}}_{\mathrm{s}}$ is the average of Y_s_(2), Y_s_(3) and Y_s_(5), then $\widehat{\widehat{\propto}}=\overline{\mathrm{Y}}-{\overline{\mathrm{Y}}}_{\mathrm{s}}$. This produced the adjusted survival probabilities at childhood. The infant and under-five mortality were estimated using the models; $\mathrm{infant}\ \mathrm{mortality}\ \mathrm{rate}=\frac{{\mathrm{q}}_0}{1-0.7\times {\mathrm{q}}_0}$ and $\mathrm{under}-5\ \mathrm{mortality}\ \mathrm{rate}=\frac{2{\mathrm{q}}_5}{2\times \left(1-{\mathrm{q}}_5\right)}$ [43].

All analysis were done using SPSS version 20.0 and Excel software.

## Results

In Table 1, the data shows that the percentage of women whose most recent under-five children died was highest among women aged 35–49 years (7.2%) and least between the age segment 25–34 years (4.7%) *p* < 0.001. The percentage of women who had lost their most recent child reduces with increasing level of education, increasing wealth quintile and level of media exposure. For instance, women who had high exposure to media had 4.5% deaths of their most recent under-five child compared to 6.7% reported by those with no media exposure.

**Table 1:** Socio-demographic characteristics of women according to deaths of under-five children

Background characteristics	Death among under-5 children (%)	Total number of children	*χ* ^2^-value	*p*-value
Mother’s Age^a^			40.10	<0.001
15–24	5.9	4746		
25–29	4.7	4843		
30–34	4.7	3830		
35–49	7.2	5097		
Highest educational level^a^			48.97	<0.001
No education	6.7	8532		
Primary	6.1	3747		
Secondary	4.3	5038		
Higher	3.2	1199		
Religion^a^			4.73	0.094
Christian	5.3	7568		
Islam	6.0	10752		
Others	5.5	200		
Ethnicity^a^			34.42	<0.001
Hausa	6.6	7234		
Igbo	6.1	1916		
Yoruba	3.5	2236		
Others	5.3	7130		
Marital status^b^			11.15	0.004
Never in union	6.9	492		
Currently in union/living with a man	5.6	17445		
Formerly in union	8.6	579		
Place of residence^a^				
Urban	4.0	6157		
Rural	6.5	12359		
Geopolitical zones^a^			48.66	<0.001
North Central	4.2	2783		
North East	6.3	3692		
North West	6.9	5816		
South East	6.6	1568		
South South	5.1	2179		
South West	3.8	2478		
Media exposure^a^			26.55	<0.001
None	6.7	6318		
Low	5.7	6013		
Medium	4.6	4607		
High	4.5	1578		

^a^Significant at 0.1%; ^b^Significant at 1.0%

Table 2 shows the results of the association between under-five survival status and maternal and child health-related characteristics. Under-five deaths was highest among women who did not attend any antenatal clinic during the child’s pregnancy (6.9%), who did not take any tetanus injection (6.8%), whose prenatal care was provided by unskilled attendant (9.1%), who were not assisted by any attendant during delivery (6.9%) and those who delivered at their homes (6.4%).

**Table 2:** Maternal and child health related characteristics according to deaths of under-five children

Background characteristics	Death among under-5 children (%)	Total number of children	*χ* ^2^-value	*p*-value
Number of antenatal visits^a^			30.62	<0.001
None	6.9	6266		
1–3	6.0	2346		
4+	4.9	9904		
Tetanus injection^a^			29.68	<0.001
None	6.8	7362		
At least 1	4.9	11154		
Sex of child^a^			8.47	0.004
Male	6.2	9347		
Female	5.2	9169		
Perceived size of baby at birth^a^			26.53	<0.001
Small	7.7	2738		
Average	5.6	7500		
Larger than average	5.1	8278		
Birth order^a^			61.86	<0.001
1st	6.2	3302		
2–3	4.3	5707		
4–5	5.0	4416		
6+	7.6	5091		
Preceding birth interval^a^			28.25	<0.001
7–23	7.2	2860		
24–35	5.8	5775		
36–59	4.6	4794		
60+	4.7	1743		
1st birth	6.4	3344		
Prenatal care provider^a^			43.25	<0.001
None	6.9	6266		
Unskilled	9.1	176		
Semi-skilled	7.0	1434		
Skilled	4.8	10640		
Delivery assistance^a^			36.52	<0.001
None	6.9	2264		
Unskilled	6.4	8385		
Semi-skilled	6.0	1092		
Skilled	4.4	6775		
Place of delivery^a^			28.060	<0.001
Home	6.4	11503		
Others	3.3	30		
Health facility	4.6	6983		

^a^Significant at 0.1%

The percentage of the most recent under-five children who had died was higher among males (6.2%) than female (5.2%) (*p* < 0.001).

**Fig. 1:**
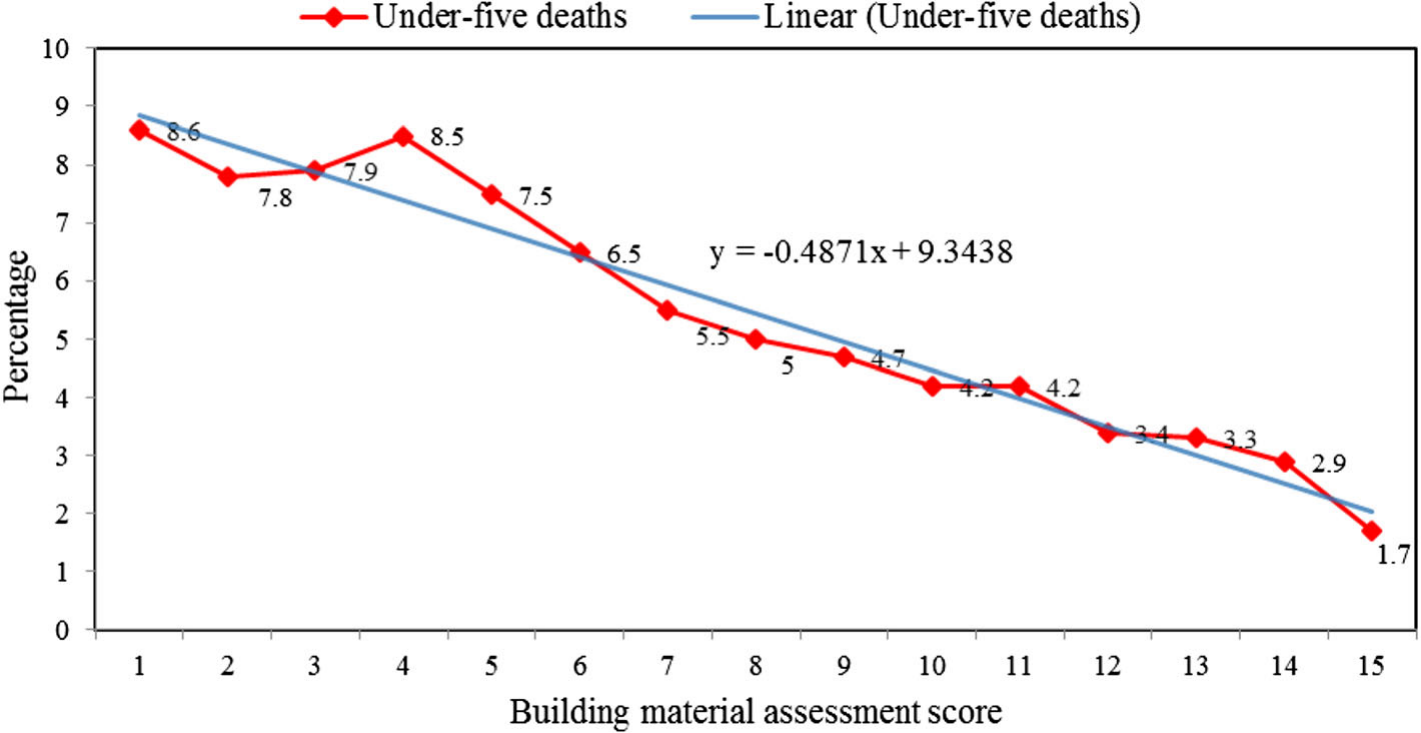
Percentage distribution of under-deaths by housing material assessment score. It presents a downward trend (slope = −0.4871) in the percentage distribution of childhood deaths according to the building materials assessment score with highest and least proportion of under-five children found among those who live in a house with 1 and 13 building assessment scores respectively

The preceding birth interval was also found to be significantly associated with under-five death with children whose their immediate senior sibling was born 7–23 months (7.2%) before their delivery experiencing highest deaths compared to those who left 24–35 months (5.8%), 36–59 months (4.6%) interval. Figure 1 shows the percentage distribution of under-deaths by building material assessment score.

The data as represented in Table 3 show that the type of cooking fuel, the source of drinking water, toilet facility and housing materials were significantly associated with childhood deaths (*p* < 0.05). However, variation exists in childhood deaths within these environmental factors. For example, women who used biomass had experienced higher under-five deaths (6.1%) than those using clean fuel (3.8%). Under-five death was lower among children of women who used improved sources (5.2%) as their drinking and cooking water than their counterparts using unimproved sources (6.3%). This pattern was similar to that of the improved and unimproved toilet facility. The results further show that women who lived in houses built with inadequate housing materials (7.1%) lost more of their under-five children compared to those who lived in houses built with adequate housing materials (4.4%) (*p* < 0.001).

**Table 3:** Environmental characteristics according to deaths of under-five children

Environmental characteristics	Death among under-5 children (%)	Total number of children	*χ* ^2^-value	*p*-value
Cooking fuel^a^			28.25	<0.001
Clean	3.8	3340		
Biomass	6.1	15176		
Source of drinking water^a^			9.64	0.002
Improved	5.2	10339		
Unimproved	6.3	8177		
Toilet facility^a^			11.47	0.001
Improved	5.1	9090		
Unimproved	6.3	9426		
Housing material^a^			50.91	<0.001
Inadequate	7.1	7035		
Moderate	5.9	3414		
Adequate	4.4	8067		

^a^Significant at 0.1%

### Multivariate results

At the multivariate level of analysis, as shown in Table 4, four models were generated. The first model included only the key independent variable (Housing materials) while the housing materials and other environmental factors were included in the second model. The third model involved the housing materials, other environmental factors and health/child-related factors while the socio-demographic and all other factors were included in the last model. The models were built in this manner so as to explore the possible interaction of the housing material and childhood mortality while other factors were used as covariates.

**Table 4:** Cox proportional hazard model of determinants of childhood mortality in Nigeria

Background characteristics	Model 1	Model 2	Model 3	Model 4
	HR (95% CIoHR)	HR (95% CIoHR)	HR (95% CIoHR)	HR (95% CIoHR)
Housing material^a^				
Inadequate	1.46 (1.24–1.71)^a^	1.36 (1.16–1.60)^a^	1.25 (1.05–1.49)^b^	1.03 (0.82–1.27)
Moderate	1.23 (1.02–1.47)^a^	1.20 (1.03–1.44)^b^	1.14 (0.94–1.37)	0.94 (0.74–1.18)
Adequate	1	1	1	1
Sex of the child				
Male		1	1	1
Female		0.82 (0.72–0.92)^a^	0.82 (0.72–0.92)^a^	0.80 (0.69–0.92)^b^
Size at birth				
Small		1	1	1
Average		0.76 (0.64–0.89)^a^	0.76 (0.64–0.90)^a^	0.81 (0.66–0.98)^c^
Larger than average		0.69 (0.52–0.81)^a^	0.70 (0.59–0.83)^a^	0.77 (0.63–0.93)^b^
Prenatal care provider				
None			1.11 (0.87–1.40)	1.07 (0.81–1.40)
Unskilled			1.78 (1.06–2.98)^b^	1.95 (0.99–3.82)
Semi-skilled			1.23 (0.97–1.57)	1.28 (0.97–1.69)
Skilled			1	1
Delivery assistance				
None			1.14 (0.79–1.66)	0.93 (0.58–1.48)
Unskilled			1.17 (0.83–1.65)	0.96 (0.62–1.48)
Semi-skilled			1.14 (0.83–1.57)	0.83 (0.55–1.23)
Skilled			1	1
Tetanus injection				
None			1.05 (0.86–1.29)	1.04 (0.82–1.32)
At least one			1	1
Number of antenatal visits				
None			1.01 (0.32–1.35)	1.02 (0.31–1.39)
1–3			1.05 (0.86–1.28)	1.05 (0.83–1.32)
4+			1	1
Toilet facility				
Improved	1	1	1	1
Unimproved	1.04 (0.92–1.19)	1.05 (0.92–1.20)	1.04 (0.91–1.19)	1.14 (0.97–1.33)
Birth order				
1st		1	1	1
2–3		3.80 (1.94–7.42)^a^	3.80 (1.94–7.42)^a^	3.61 (1.46–8.88)^b^
4–5		4.44 (2.21–8.89)^a^	4.44 (2.21–8.89)^a^	3.80 (1.49–9.68)^b^
6+		6.47 (3.24–12.91)^a^	6.47 (3.24–12.91)^a^	4.93 (1.92–12.60)^b^
Cooking fuel				
Clean	1	1	1	1
Biomass	1.31 (1.06–1.62)^a^	1.22 (0.99–1.51)^b^	1.16 (0.94–1.44)	0.81 (0.56–1.18)
Place of delivery				
Home			1	1
Others			0.94 (0.68–1.29)	0.71 (0.09–5.11)
Health facility			0.47 (0.65–3.44)	1.11 (0.74–1.66)
Age group				
15–24				1
25–29				0.97 (0.76–1.22)
30–34				0.95 (0.71–1.26)
35–39				1.20 (0.89–1.62)
Source of drinking water				
Improved	1	1	1	1
Unimproved	1.05 (0.93–1.20)	1.04 (0.92–1.19)	1.02 (0.90–1.17)	1.02 (0.88–1.18)
Geopolitical zones				
North Central				1.13 (0.69–1.84)
North East				1.57 (0.96–2.55)
North West				1.85 (1.11–3.05)^c^
South East				2.54 (0.87–7.37)
South South				1.29 (0.77–2.13)
South West				1
Preceding birth interval				
7–23		1	1	1
24–35		0.81 (0.68–0.96)^b^	0.81 (0.68–0.97)^b^	0.82 (0.67–0.99)^c^
36–59		0.64 (0.53–0.77)^a^	0.65 (0.53–0.78)^a^	0.64 (0.51–0.79)^a^
60+		0.69 (0.53–0.89)^a^	0.70 (0.54–0.90)	0.57 (0.41–0.79)^b^
1st birth		4.39 (2.23–8.63)^a^	4.56 (2.31–8.99)^a^	3.91 (1.57–9.70)^b^
Place of Residence				
Urban				1
Rural				1.23 (0.87–2.54)
Highest educational level				
None				1.73 (0.85–3.52)
Primary				1.94 (0.96–3.89)
Secondary				1.51 (0.76–2.96)
Tertiary				1
Ethnicity				
Hausa/Fulani				0.99 (0.79–1.25)
Igbo				0.69 (0.26–1.79)
Yoruba				0.82 (0.46–1.45)
Others				1
Media exposure				
None				1
Low				0.97 (0.82–1.14)
Medium				1.05 (0.82–1.33)
High				1.01 (0.62–1.61)
Marital status				
Never in union				1
Currently in union				0.65 (0.42–0.99)^c^
Formerly in union				1.01 (0.59–1.71)

*HR* hazard ratio, *CIoHR* confidence interval of hazard ratio

^a^Significant at 0.1%; ^b^Significant at 1.0%; ^c^Significant at 5.0%

The data revealed that the type of housing materials was significantly associated with childhood mortality across the first three models. For instance, in model 1, the hazard ratio of childhood mortality was 1.23 (C.I = 1.24–1.71, *p* < 0.001) and 1.46 (C.I = 1.02–1.47, *p* < 0.001) higher among children who lived in houses built with moderate and inadequate housing materials respectively than those living in houses built with adequate housing materials. This pattern was found across the models but in the last model, housing materials were found to be insignificantly associated with childhood mortality showing that socio-demographic factors are strongly important in the relationship between childhood mortality and housing materials. The identified predictors of childhood mortality are; the size of the child at birth, preceding birth interval, prenatal care provider, place of residence and education. The likelihood of childhood mortality was 0.82 (C.I = 0.72–0.92; *p* < 0.001) lower among females than males, among children who were average (HR = 0.76; C.I = 0.64–0.90) or larger (HR = 0.71; C.I = 0.60–0.84, *p* < 0.001) than average in size at birth than those who were small. The data further show that childhood mortality hazard was 1.35 (C.I = 1.13–1.62, *p* < 0.001) more in the rural areas than the urban.

#### Infant and under-five mortality rates by housing material type

In Table 5, the data show that at different levels of housing materials type (inadequate, moderate and adequate), the refined childhood mortality probability estimate increases as the age of child increases. For instance, among the group of women who live in houses built with inadequate housing material, the refined childhood mortality probability increases from 0.0778 among infants to 0.1613 among young adults. The data further show that infant mortality probability increases considerably as the level of housing materials type increases.

**Table 5:** Estimated smoothed childhood mortality probability according to housing materials type, 2013 Nigeria demographic and health survey

Age group	Pi	Di	Age x	*l*(x)_Es_	*l*(x)_Br_	Y(x)	Y(s)	Adj. Y(x)	Adj. q(x)	Ref. period
Housing material = IN; Mortality level =16.29; $\overline{Y(x)}$ = −1.02647; $\overline{Y(s)}$ = −0.6573; $\widehat{\widehat{\propto}}$ = −0.3692; β = 1.0										
15–19	1.24	0.1156	1	1.00015	0.91303	−1.1756	−0.8670	−1.2362	0.0778	2010.4
20–24	2.13	0.1426	2	0.88676	0.89479	−1.0703	−0.7152	−1.0844	0.1026	2008.9
25–29	3.77	0.1793	3	0.82322	0.88651	−1.0278	−0.6552	−1.0244	0.1142	2007.8
30–34	5.36	0.1985	5	0.78822	0.87682	−0.9813	−0.6015	−0.9707	0.1255	2007.1
35–39	6.90	0.2222	10	0.75241	0.86552	−0.9309	−0.5498	−0.9190	0.1373	2006.6
40–44	8.01	0.2362	15	0.73754	0.85705	−0.8955	−0.5131	−0.8823	0.1462	2005.5
45–49	9.43	0.2711	20	0.70206	0.84449	−0.8460	−0.4551	−0.8243	0.1613	2003.0
Housing material = MO; Mortality level =16.98; $\overline{Y(x)}$ = −1.08753; $\overline{Y(s)}$ = −0.6573; $\widehat{\widehat{\propto}}$ = −0.4302; β = 1.0										
15–19	1.25	0.1476	1	1.005	0.92114	−1.2289	−0.867	−1.2972	0.0695	2010.3
20–24	2.08	0.1309	2	0.89822	0.90553	−1.1301	−0.7152	−1.1454	0.0919	2008.8
25–29	3.53	0.1657	3	0.83811	0.89828	−1.0891	−0.6552	−1.0854	0.1024	2007.7
30–34	5.13	0.1772	5	0.81199	0.88961	−1.0434	−0.6015	−1.0317	0.1127	2006.9
35–39	6.70	0.2012	10	0.77681	0.87945	−0.9936	−0.5498	−0.9800	0.1235	2006.3
40–44	7.54	0.1895	15	0.79033	0.87179	−0.9585	−0.5131	−0.9433	0.1316	2005.3
45–49	9.04	0.2052	20	0.77545	0.86030	−0.9089	−0.4551	−0.8853	0.1455	2002.8
Housing material = AD; Mortality level =18.68; $\overline{Y(x)}$ = −1.2556; $\overline{Y(s)}$ = −0.6573; $\widehat{\widehat{\propto}}$ = −0.5983; β = 1.0										
15–19	1.22	0.0921	1	1.01869	0.93998	−1.3756	−0.8670	−1.4653	0.0507	2010.0
20–24	1.79	0.1044	2	0.92512	0.93007	−1.2939	−0.7152	−1.3135	0.0674	2008.3
25–29	2.74	0.1127	3	0.89233	0.92520	−1.2576	−0.6552	−1.2535	0.0754	2007.1
30–34	3.90	0.1151	5	0.87886	0.91914	−1.2153	−0.6015	−1.1998	0.0832	2006.4
35–39	5.24	0.1418	10	0.84323	0.91172	−1.1674	−0.5498	−1.1481	0.0914	2006.0
40–44	6.33	0.1495	15	0.83494	0.90606	−1.1332	−0.5131	−1.1114	0.0977	2005.1
45–49	7.44	0.1551	20	0.83069	0.89718	−1.0831	−0.4551	−1.0534	0.1084	2002.7
Housing material = Total; Mortality level =16.95; $\overline{Y(x)}$ = −1.08477; $\overline{Y(s)}$ = −0.6573; $\widehat{\widehat{\propto}}$ = −0.4275; β = 1.0										
15–19	1.24	0.1184	1	1.00248	0.92078	−1.2265	−0.8670	−1.2945	0.0699	2010.4
20–24	2.08	0.1310	2	0.89781	0.90506	−1.1274	−0.7152	−1.1427	0.0923	2008.8
25–29	3.53	0.1613	3	0.84245	0.89777	−1.0863	−0.6552	−1.0827	0.1029	2007.7
30–34	5.03	0.1741	5	0.81548	0.88905	−1.0406	−0.6015	−1.0290	0.1133	2006.9
35–39	6.55	0.1975	10	0.78121	0.87884	−0.9907	−0.5498	−0.9773	0.1241	2006.3
40–44	7.64	0.2065	15	0.77185	0.87115	−0.9556	−0.5131	−0.9406	0.1323	2005.2
45–49	8.85	0.2358	20	0.74232	0.85961	−0.9060	−0.4551	−0.8826	0.1462	2002.7

*P(i)* Parity, *D(i)* proportion of children dead, *q(x)* probability of dying, *l(x)* probability of surviving, *MHCSAI* maternal health care service access index, *ES* estimated, *Br* brass, *Ref.* reference, $\overline{Y(x)}$ logitl(x), $\overline{Y(s)}$ logitl(s), $\overline{\overline{\propto}}$ Mean of estimated parameter ∝, *IN* inadequate, *MO* moderate, *AD* adequate

**Fig. 2:**
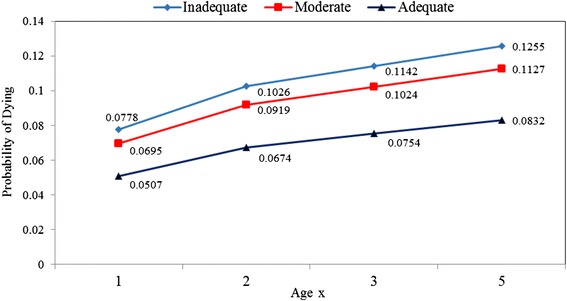
Childhood probability of dying at age x according to housing materials. It provides the pattern of the probability of dying at ages 1, 2, 3 and 5. There was a consistent increasing pattern in the probability of dying from ages 1 to 5 years and maternal housing materials type

**Fig. 3:**
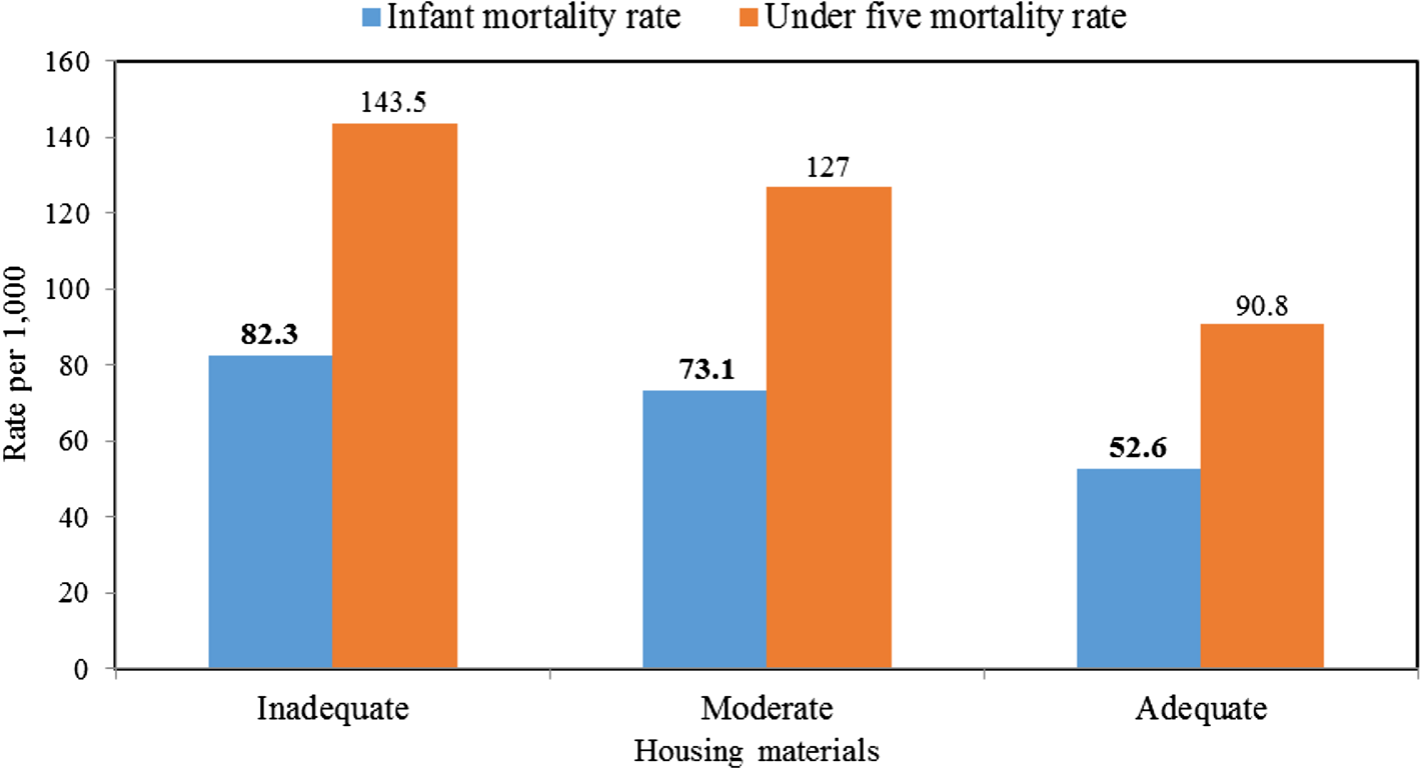
Infant and Under-five mortality rates according to housing materials type. The data show that infant mortality rate reduces from 82.3 per 1000 live birth among women who live in houses built with inadequate housing materials to 52.6 per 1000 live birth among those live in houses built with adequate housing materials. This pattern was also observed for under-five mortality across the housing materials categories

For instance, the probability of dying at exact age 1 year was highest among women who live in houses built with inadequate housing materials (0.0778) and least among their counterparts whose houses were built with adequate materials (0.0507). Figure 2 shows childhood probability of dying at age x according to housing materials. The estimated infant and under-five mortality rates which were obtained from the smoothed childhood mortality probabilities in Table 5 are displayed in Fig. 3.

## Discussion

Housing and health are essential for human wellness and quality of life [44]. Poor housing conditions have been seen to represent a stern environmental health threat that is preventable [45]. Our study seeks to understand the effect of housing materials on under-five mortality in Nigeria.

Findings from our study show that the risk of dying before the age of 5 years was higher among children who lived in houses built with inadequate housing materials than those in moderate and adequate housing materials. This results gave credence to other studies that established an association between ill health and poor housing conditions [46–49]. The quality of the materials with which a building is built is mostly linked with the durability of the building and the health of its occupants [9]. Existing literature indicates general health improvements with improved housing conditions [50–52]. Izugbara [53], in his study, reported that households with floors made of mud or sand are more likely to experience under-5 mortality than households with cement residence floors.

Also, recent epidemiological studies revealed that living in houses built with inferior materials could result in chronic diseases [54, 55]. Researchers assert that houses built with mud or sand are usually not durable and are prone to damp conditions which encourage the growth of moulds in an indoor environment [35]. Exposure to damp and mouldy housing could lead to the occurrence of asthma and other chronic respiratory infections in children [54, 55]. Asthma is the most common non-communicable disease among children, affecting 235 million people globally [56].

Moreover, damp buildings produce cracks on walls which provide a fostering environment for cockroaches, moulds, mites and respiratory viruses, all of which promote respiratory disease pathogenesis [35, 57]. Cockroaches can effect allergic sensitisation and have been seen to trigger asthma occurrence in urban areas. Children presenting with asthma who are sensitised and exposed to cockroaches are at higher risk of hospitalisation [58]. Also, exposure to pollutants such as formaldehyde and volcanic organic compounds, which are emitted from building materials have been associated with asthma, and bronchial obstruction in the first 24 months of life [59, 60]. Our study revealed that women who used biomass, water from unimproved sources and unimproved toilet facilities had experienced higher under-five deaths than those using clean fuel, improved water sources and improved toilet facilities respectively. Indeed, the use of solid fuels in homes have been reported to be the leading single environmental cause of ill health [61–63]. Previous studies have indicated that children living in homes using solid fuels for cooking are at a greater risk of dying from acute respiratory illnesses [64–66]. According to the WHO [25], about half of all deaths among under-five children in the year 2014 was caused by acute lower respiratory infections triggered by the use of solid fuels.

Sourcing water from unimproved sources and poor sanitation practices have been implicated in the death of a child every 15 s from diarrhea disease [67]. According to the WHO, diarrhea, annually accounts for 1.7 million morbidities and 760, 000 under-five children’s deaths globally [68]. It remains the second leading cause of death among children under-five globally [69].

This study further revealed that child and maternal characteristics such as sex the child, the size of the child at birth, preceding birth interval, prenatal care provider, place of residence and education, play a significant role in under-five mortality. This corroborated earlier findings from Nigeria and other places in the direction of these associations between under-five deaths and child and maternal characteristics [53, 64, 65, 70–77].

### Policy implication

Globally, scientific evidence on the associations between housing and health has grown considerably in recent years. This information has been used as preventive measures that are linked to better housing condition and human health. From a policy point of view, there is a need for formulation of guidelines on healthy housing by the government and other relevant stakeholders to assist in averting a wide range of preventable illnesses that are often associated with poor housing conditions. This position has earlier been echoed at the international consultation of 40 specialists drafted from 18 countries by the WHO on 13–15 October 2010 in Geneva [78]. In this regard, a national standard on “healthy housing would enable action that is scientifically based, and protects and advances the health of under-five children in households” [78]. The existing national housing policy that sees housing as a determinant of health and social stability should be strengthened in Nigeria. However, the most healthy and safest house will not support the health of its inhabitants if not maintained and used in a manner that promotes sound health. Therefore, individuals should be advised to use good housing materials while building their houses. Even the poor can get good housing materials from the little resources they have if properly managed.

### Limitation

The major shortcoming of this study is the cross-sectional nature of the data where information on housing materials was based on verbal reporting instead of the use of check listing approach which could have revealed the true state of housing condition in the study area. Although this limitation was minimized since data were collected at the individual homes, the data collectors were able to assess the structures and match their observations with the report from the respondents. Also, the reporting of under-five children’s death by women may include omission of few dead children. Culturally, in sub-Saharan Africa, people dislike talking about their dead children as they see this as a bad event they don’t want to remember. However, in this study, we used an indirect method that accounts for such limitation for the estimation of childhood mortality across the classes of housing materials.

## Conclusion

The death of under-five children in the face of various factors remains a problem in Nigeria as the estimated infant and under-five mortality rates in this study are high. Living in houses built with inadequate housing materials promoted under-five mortality in Nigeria. Also, housing materials, environmental factors in addition to maternal and childhood characteristics were associated with childhood mortality. Housing materials remain a predictor of under-five mortality when environmental, child’s characteristics and health-related factors were used as covariates. However, amidst all factors, housing materials may not be so important, instead maternal education, place of residence, sex, the size of the child at birth, prenatal care provider, birth order and preceding birth interval are the key predictors of deaths among under-five children in Nigeria. The existence of under-five mortality inequalities across different areas, regions and socio-economic groups is also well documented. Various reforms and policies have been instituted by the government and relevant stakeholders to address the high rate of under-five mortality in Nigeria but these are yet to yield the expected results. Improvements in housing conditions and in the continuum of maternal and child health will play a significant role in reducing the burden of under-five mortality in Nigeria.

## Abbreviations

ARIs: Acute respiratory infections
C.I: Confidence interval
CIoHR: Confidence interval of hazard
DHS: Demographic and health surveys
HR: Hazard ratio
MDGs: Millennium development goals
SDGs: Sustainable development goals
U5M: Under-five mortality
UNDESA: United Nations Department of Economic and Social Affairs
UNICEF: United Nations Children’s Fund
VPD: Vaccines preventable diseases
WHO: World Health Organisation
